# Supplementary material for: Peroxisomal Localization of a Truncated HMG-CoA Reductase under Low Cholesterol Conditions
Source: Biomolecules. 2024 Feb 19;14(2):244. doi: 10.3390/biom14020244 (PMC10886633; doi:10.3390/biom14020244)
Supplement: Supplementary file 1 [file biomolecules-14-00244-s001.zip › biomolecules-2852771-supplementary.pdf]

Supplementary Material

Peroxisomal Localization of a Truncated HMG-CoA Reductase under Low Cholesterol Conditions

Jianqiu Wang, Markus Kunze, Andrea Villoria-González, Isabelle Weinhofer and Johannes Berger

List of supplementary materials:

- Supplementary Figure S1. The monoclonal anti–HMGCR antibody (anti-HMGCR-A9) stains predominantly the ER.
- Supplementary Figure S2. Specificity controls for the antibodies in THP-1 cells.
- Supplementary Figure S3. Unprocessed full-size images of Figure 3.
- Supplementary Figure S4. Independent density gradient and western blot analysis of peroxisomal HMGCR in differentiated THP-1 cells grown under low cholesterol conditions and lovastatin treatment.

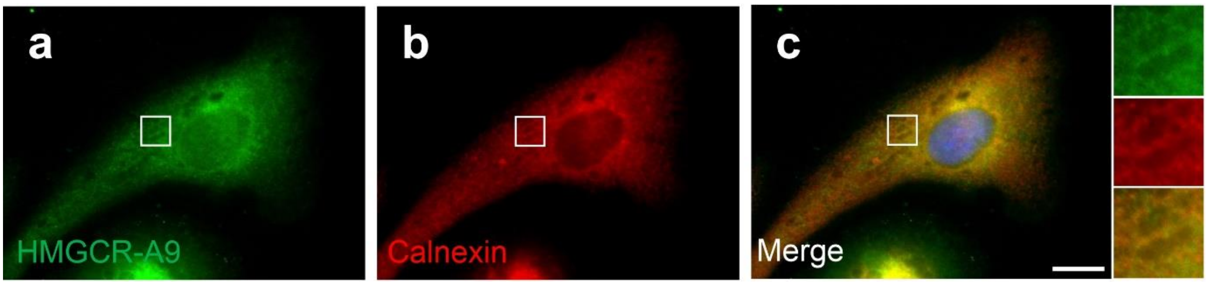

Supplementary Figure S1. The monoclonal anti–HMGCR antibody (anti-HMGCR-A9) stains predominantly the ER. (a-c) Cells of the human monocytic cell-line THP-1 were differentiated using 40 ng/ml PMA and cultivated in lipid-depleted medium for 7 days, with 5 µM lovastatin added for the last 16 hours to increase endogenous HMG-CoA reductase (HMCGR) protein levels. The subcellular distribution of HMGCR was investigated by immunofluorescence microscopy: (**a**) Monoclonal α-HMGCR-A9 (green), (**b**) polyclonal α-Calnexin (red, labeling the ER) and (**c**) overlay. The co-localization demonstrates that the monoclonal HMGCR-A9 antibody detects HMGCR in the ER. Scale bar, 20 µm.

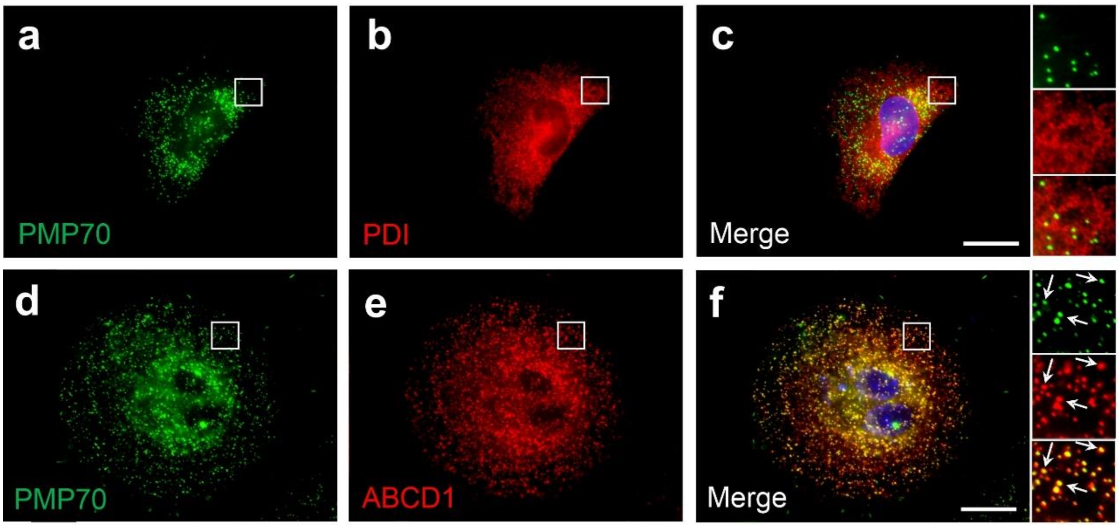

Supplementary Figure S2. Specificity controls for the antibodies in THP-1 cells. (**a-f**) Immunofluorescence microscopic analysis of PMA-differentiated THP-1 cells (7 days) under low cholesterol condition. The signals obtained for the peroxisomal marker protein, PMP70 (green) and the ER marker protein, PDI (red), showed no co-localization (**a-c**); while PMP70 (green) and another peroxisomal marker protein, ABCD1 (red), showed very strong co-localization mutually confirming their peroxisomal localization (**d-f**). White arrows: punctate, peroxisomal staining pattern. Scale bars, 20 μm.

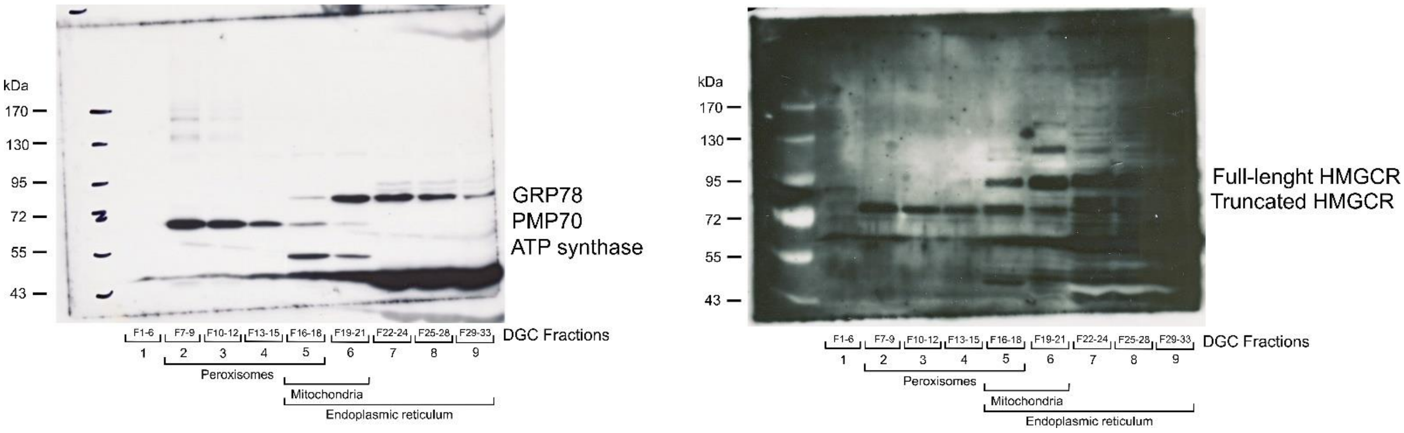

Supplementary Figure S3. Unprocessed full-size images of Figure 3. In differentiated THP-1 cells grown under low cholesterol condition and lovastatin treatment a truncated form of HMGCR was found in peroxisome enriched fractions obtained by density gradient centrifugation (DGC). 40 flasks of THP-1 cells were differentiated and incubated in lipid depleted medium for 7 days. 5 μM lovastatin was provided for the last 16 h before harvesting (low cholesterol condition). DGC was used to enrich organelles and separate fractions with different composition according to their density. Fractions were then pooled and western blots were per-formed using α-GRP78, α-PMP70 and α-ATP synthase antibodies to identify fractions enriched in ER (fraction 16-33, lane 5-9), peroxisome (F7-18, lane 2-5) and mitochondria (F16-21, lane 5-6), respectively. Whereas full length HMGCR with a size of about 97 kDa was found in fractions enriched in the ER (Lanes 5-9), a truncated form HMGCR with a size of about 76 kDa was found in peroxisome enriched fractions (Lane 2-5). The size of the molecular weight marker is indicated on the left side.

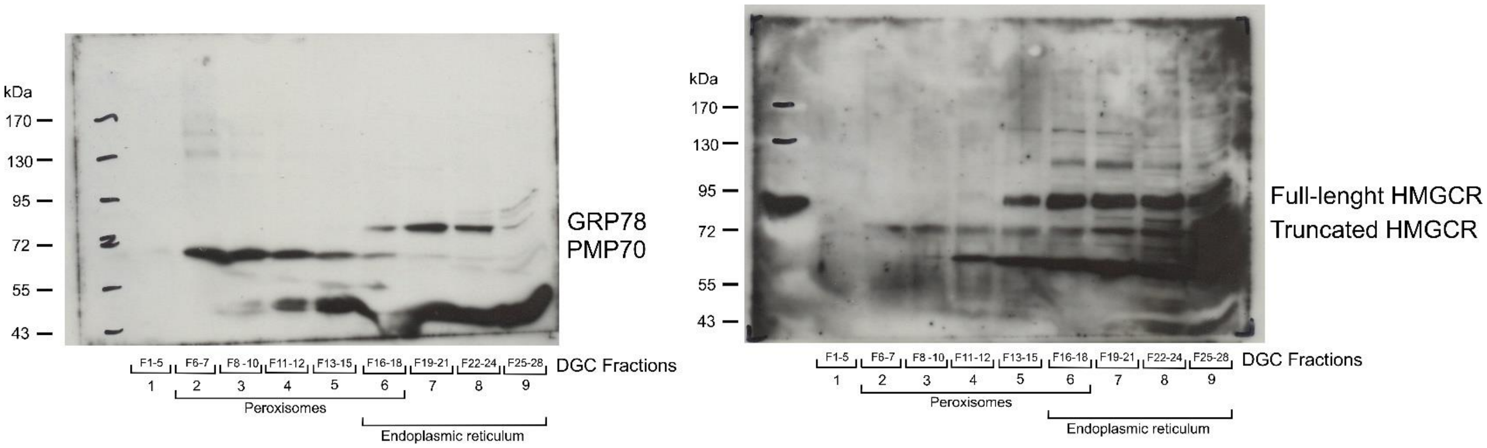

Supplementary Figure S4. Independent density gradient and western blot analysis of peroxisomal HMGCR in differentiated THP-1 cells grown under low cholesterol conditions and lovastatin treatment. As in Figure 3 and Supplementary Figure 3, a shorter (~76 kDa) HMGCR fragment was detected also in the PMP70-positive gradient fractions containing the peroxisomes, whereas the full-length HMGCR was confined to the GRP78-positive, ER-containing microsomal fractions.
